# Molecular Dynamic Simulations to Probe Stereoselectivity of Tiagabine Binding with Human GAT1

**DOI:** 10.3390/molecules25204745

**Published:** 2020-10-16

**Authors:** Sadia Zafar, Ishrat Jabeen

**Affiliations:** Research Center for Modeling and Simulation (RCMS), National University of Sciences and Technology (NUST), Islamabad 44000, Pakistan; sadia.zafar@rcms.nust.edu.pk

**Keywords:** molecular dynamics, GAT1 inhibitors, tiagabine stereoisomers, GABA reuptake

## Abstract

The human gamma aminobutyric acid transporter subtype 1 (hGAT1) located in the nerve terminals is known to catalyze the neuronal function by the electrogenic reuptake of γ-aminobutyric acid (GABA) with the co-transport of Na^+^ and Cl^−^ ions. In the past, there has been a major research drive focused on the dysfunction of hGAT1 in several neurological disorders. Thus, hGAT1 of the GABAergic system has been well established as an attractive target for such diseased conditions. Till date, there are various reports about stereo selectivity of –COOH group of tiagabine, a Food and Drug Administration (FDA)-approved hGAT1-selective antiepileptic drug. However, the effect of the stereochemistry of the protonated –NH group of tiagabine has never been scrutinized. Therefore, in this study, tiagabine has been used to explore the binding hypothesis of different enantiomers of tiagabine. In addition, the impact of axial and equatorial configuration of the–COOH group attached at the meta position of the piperidine ring of tiagabine enantiomers was also investigated. Further, the stability of the finally selected four hGAT1–tiagabine enantiomers namely entries 3, 4, 6, and 9 was evaluated through 100 ns molecular dynamics (MD) simulations for the selection of the best probable tiagabine enantiomer. The results indicate that the protonated –NH group in the *R*-conformation and the –COOH group of Tiagabine in the equatorial configuration of entry 4 provide maximum strength in terms of interaction within the hGAT1 binding pocket to prevent the change in hGAT1 conformational state, i.e., from open-to-out to open-to-in as compared to other selected tiagabine enantiomers 3, 6, and 9.

## 1. Introduction

γ-aminobutyric acid (GABA) is the most abundant inhibitory neurotransmitter in the mammalian central nervous system (CNS) that contributes to the regulation of several physiological processes such as plasticity, learning, and memory [1]. The major neuronal regulator of GABA i.e., GABA transporter 1 (GAT1), helps in maintaining the extracellular GABA concentration required for the activation of postsynaptic GABA receptors for fast inhibitory neurotransmission [2]. After inhibitory neurotransmission, the major proportion of extra GABA from the synaptic cleft is reverted to presynaptic neurons through the human gamma aminobutyric acid transporter subtype 1 (hGAT1) reuptake process [3,4]. With regard to GAT1’s function in GABA regulation, abnormal hGAT1 neurotransmission has been associated with low GABA levels in the synaptic cleft, which may implicate several neurological disorders such as Alzheimer’s disease [5], schizophrenia [6,7], Parkinson’s disease [8], and epilepsy [9]. Thus, to amplify the GABA-mediated inhibitory neurotransmission/GABA activity in the CNS, the most widely utilized approach is the inhibition of GABA reuptake by locking hGAT1 in its open-to-out conformation [4,10]. Hence, among all the three distinct conformations of hGAT1 during the translocation cycle, i.e., open-to-out (mediating the co-transport of two Na^+^ and one Cl^−^ ions along with the substrate GABA from the extracellular space), occluded-out (representing the sealed Na^+^/Cl^−^ ions and GABA in the binding pocket of hGAT1), and open-to-in (releasing ions and GABA into the intracellular space) conformations, the open-to-out conformation is the most targeted conformation to block the reuptake of extra GABA from the extracellular space thereby normalizing the synaptic GABA concentration [11,12,13].

**Figure 1:**
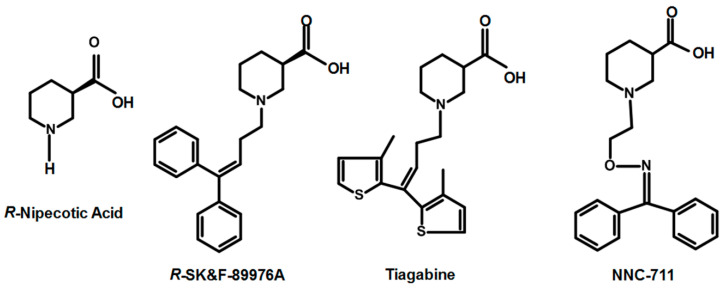
Chemical structures of well-known inhibitors of the human gamma aminobutyric acid transporter subtype 1 (hGAT1).

The determination of the crystal structures of *Aquifex aeolicus’* leucine transporter (*Aa*LeuT) [14] and *Drosophila melanogaster’s* dopamine transporter (dDAT) [15] provided the possibility of the first structure-based ligand docking and simulation in hGAT1. Since 1950s, several different functional groups have been introduced to the hGAT1 inhibitors in order to improve their selectivity and affinity. Until now, nipecotic acid (polar, zwitterionic GABA analog) and its subsequent synthesized derivatives are employed to inhibit in vitro hGAT1 activity [16,17,18]. The general architecture of hGAT1 inhibitors has a common pattern of attachment of lipophilic chain to the parent molecule (e.g., nipecotic acid) followed by the substitution of aromatic moieties as in case of two well-known hGAT1 inhibitors tiagabine [19] and SKF-89976A [20], of which tiagabine is the only approved antiepileptic FDA drug [21]. Previous studies illustrate that the *R*-enantiomers of hGAT1 inhibitors, e.g., *R*-nipecotic acid and *R*-SK&F-89976A (Figure 1), are known to have better hGAT1 inhibitory activity in comparison to *S*-enantiomers [22].

However, the impact of stereoisomerism (*R*/*S* configuration) of the protonated –NH group of the polar moiety (e.g., piperidine, pyrrolidine, or azetidine ring) along with the orientation of aromatic moieties attached to the linker chain of hGAT1 inhibitors on biological activity (IC_50_) has not been determined yet. Therefore, the current study explores the binding hypothesis of *R*/*S* conformations of the –NH group that may provide a starting point for the design of a new set of selective inhibitors of hGAT1 in neurological disorders. The finally selected binding hypothesis of tiagabine distereoisomers was further cross validated with the stereoisomers of another known inhibitor of GAT1, i.e., NNC-711 (Figure 1).

## 2. Results and Discussion

### 2.1. Docking and Clustering of R- and S-enantiomers of Tiagabine in hGAT1

Overall, binding solutions of both *R*- and *S*-enantiomers of tiagabine obtained in docking protocol I shared similar binding positions at transmembrane (TM) segments 1a, 1b, 6a, 6b, and 10 of hGAT1. However, binding conformations of the –NH group, –COOH group, and thiophene rings of *R*- and *S*-tiagabine were different. Generally, the unwound regions between the TM segments 1a, 1b, 6a, and 6b are known to majorly involve in the upholding of 2Na^+^/1Cl^−^ ions and ligands in hGAT1 [4,11,23]. Overall, 10 out of the total generated 40 binding solutions of *R*- and *S*-tiagabine (Supplementary Materials, Figure S1) that showed distinct interaction patterns and the best G_score_ were selected for further comparison with respective constraint docking solutions.

**Figure 2:**
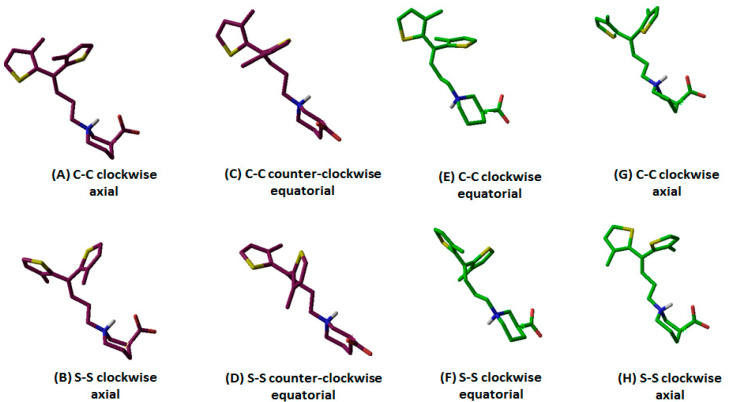
(**A**–**D**) *R-* (maroon) and (**E**–**H**) *S-* (green) configured –NH group of tiagabine enantiomers further classified on the basis of different orientations of thiophene rings and –COOH group.

Out of 10 selected hGAT1–tiagabine complexes, four belonged to *R*-configured and the other six to *S*-configured –NH group of tiagabine enantiomers. As an example, the orientation and associated terminology of tiagabine enantiomers are shown in Figure 2, where *R*-enantiomers of tiagabine are shown with in maroon and *S*-enantiomers are represented with green. Moreover, the term C–C represents the twisting of methyl groups (clockwise and counterclockwise) attached to the thiophene rings, whereas S–S represents the orientation (clockwise and counterclockwise) of sulfur atoms in thiophene rings. Table 1 represents a list of all 10 hGAT1–tiagabine enantiomers for ligand–protein interaction analysis.

Briefly, in the case of *R*-configured tiagabine, the twisting of thiophene rings in the clockwise orientation (in which methyl groups were toward the observer’ eye) along with the axial conformation of the –COOH group was declared as “*R*-configured C–C clockwise axial” tiagabine conformation (Figure 2A, Table 1; entry 1,) whereas when sulfur atoms in thiophene rings were toward the observer’s eye, then that conformation was termed as “*R*-configured S–S clockwise axial” tiagabine conformation (Figure 2B, Table 1; entry 2). Moreover, all the four *R*-configured tiagabine conformations represent enantiomers of the four *S*-configured tiagabine conformations. However, two additional conformations belonging to the *S*-configured tiagabine including “C–C clockwise equatorial” (Figure 2E, Table 1; entry 9) and “S–S clockwise equatorial” were also observed (Figure 2F, Table 1; entry 10). Furthermore, similar docking conformations with the lowest G_score_ values were selected in the case of constraint docking (protocol II) as shown in Table 2. As for Glide SP, scores of −10 or lower usually represent good binding; therefore, at this point, G_score_ in both of the docking scenarios could not be considered either bad or good.

**Figure 3:**
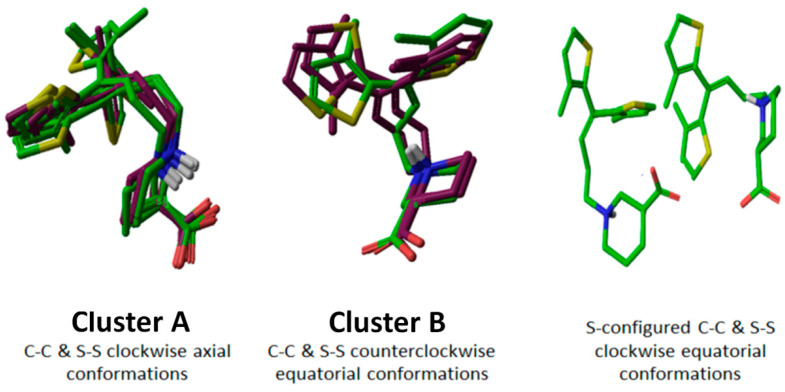
Clustering of *R*- and *S*-configured tiagabine enantiomers. Superimposition showed a similar orientation pattern in cluster A and B*_unconstraint_;* however, two *S*-configured C–C/S–S clockwise equatorial*_unconstraint_* tiagabine enantiomers with different conformation patterns in hGAT1 are also presented.

On the basis of conformational overlap, *R*- and *S*-configured tiagabine (Table 1) resulted in two major clusters of poses, i.e., C–C/S–S clockwise axial (cluster A*_unconstraint_*) and C–C/S–S counterclockwise equatorial (cluster B*_unconstraint_*). Along with cluster A and B*_unconstraint_*, two *S*-configured C–C/S–S clockwise equatorial unconstraint tiagabine enantiomers (entry 9 and 10 in Table 1) with different conformational patterns in the hGAT1 binding pocket were also selected for further ligand–protein interaction analysis as shown in Figure 3.

### 2.2. Ligand–Protein Interaction Analysis of Unconstraint Docking Solutions

Briefly, in the case of cluster A*_unconstraint_,* the axial –COOH group of tiagabine enantiomers showed hydrogen bond interactions with –NH of G65 (Table 3). It is generally known that the attachment of *R*/*S*–COOH group to the polar moiety of hGAT1 antagonists results in the formation of a hydrogen bond with the G65(–NH) of the hGAT1 [23]. Additionally, a distance of 3.71–4.00 Å between –OH of Y140 and –COOH was observed as shown in Figure S2A from Supplementary Materials, Table 3 (with the exception of entry 6 of Table 1 where hydrogen bonding was observed between –OH of Y140 and –COOH). Moreover, the axial –COOH of tiagabine enantiomers and Na1 were separated at a distance range of approximately 2.32–2.45 Å (Supplementary Materials, Figure S2A). Previously, Ben-Yona and Kanner highlighted the importance of Na1 by the establishment of capacitative transient currents due to the flux of Na1 within the binding pocket of hGAT1 [24]. In another study, Skovstrup et al. [25], observed a continuous interaction switch between F294(O) and S295(O) of hGAT1 with the protonated –NH group of tiagabine during an MD simulation, which is in line with the current study where hydrogen bond interaction was observed between S295(O) and the protonated –NH group of a few of the tiagabine enantiomers. However, the maximum observed distance was approximately 3.33–3.62 Å (Table 3).

Cluster B*_unconstraint_* occupied a similar position in the hGAT1 binding cavity to that of cluster A*_unconstraint_*. Peculiarly, the equatorial –COOH group showed an electrostatic interaction with the Na1 at a distance of 2.53–2.60 Å and hydrogen bond interactions with –NH of G65 and –OH of Y140 (Supplementary Materials, Figure S2B). However, in a few binding poses a distance of 3.11–3.50 Å was observed between –OH of Y140 and –COOH group (Table 3). Furthermore, the backbone carbonyl oxygen moiety of F294 showed hydrogen bond interaction with the protonated –NH group of a few of the tiagabine enantiomers while the rest were separated at a distance of 3.86–4.02 Å. However, the only exception was *S*-configured S–S clockwise equatorial enantiomer (Table 1, entry 10) in which the distances between –COOH group and –OH of Y140, –NH of G65, and Na1 were 5.7, 3.99, and 4.05 Å, respectively (Supplementary Materials, Figure S2C). In addition, the approximate distance between F294(O) and the protonated –NH group of tiagabine was also increased to 4.23 Å.

### 2.3. Ligand–Protein Interaction Analysis of Constraint Docking Solutions

In constraint docking, only those binding poses were sampled occupying positions of cluster A*_unconstraint_*, B*_unconstraint_*, and positions of entry 9 and 10 (as in case of unconstraint docking) and also designated as cluster A*_(hydrophobic constraint, Y140 constraint, both constraints)_*, B*_(hydrophobic constraint, Y140 constraint, both constraints)_*, and positions of additional two enantiomers *_(hydrophobic constraint, Y140 constraint, both constraints)_*, i.e., entry 9 and 10.

The application of the hydrophobic region constraint revealed a similar interaction pattern as explained for clusters A*_unconstraint_* and B*_unconstraint_* in unconstrained docking as shown in Table 3. Briefly, –COOH groups showed hydrogen bond interaction with G65 and were present at a distance of 2.33–2.60 Å from Na1 (Table 3). However, the distance of the protonated –NH group with S295 in cluster A*_hydrophobic constraint_* was 3.21–3.50 Å (Supplementary Materials, Figure S3A) and that with F294 in cluster B*_hydrophobic constraint_* was 3.84–3.99 Å (Supplementary Materials, Figure S3B), hereby resulting in the disruption of hydrogen bond interaction. Furthermore, the tiagabine enantiomers in cluster A*_hydrophobic constraint_* possessing an axial –COOH group showed a distance of approximately 4.43 Å from the –OH of Y140 (Supplementary Materials, Figure S3A) whereas, equatorial –COOH groups of cluster B*_hydrophobic constraint_* were separated at a distance of 2.46–3.09 Å from –OH of Y140 (Supplementary Materials, Figure S3B). Similarly, in case of *S*-configured C–C/S–S clockwise equatorial tiagabine enantiomers (entries 9 and 10 of Table 1) only hydrogen bond interaction between –COOH and –NH of G65 was observed (Table 3).

In hydrogen bond constraint at Y140, the binding solutions of tiagabine showed a maximum distance of 3.22–6.02 Å between –COOH of tiagabine enantiomers and Na1 (Supplementary Materials, Figure S3C,D). This showed that the hydrogen bonding constraint could result in the loss of coordination with Na1, whose binding with acid group (i.e., –COOH) of the ligand (tiagabine) is crucial for translocation [26]. Moreover, none of the enantiomers in both clusters (A and B of Y140 constraint) along with entries 9 and 10 of (Table 1) showed interaction with the protonated –NH group of tiagabine (Table 3) because the distance range for the protonated –NH group and S295 (O) in cluster A*_Y140 constraint_* was 4.89–5.18 Å (Supplementary Materials, Figure S3C) and that with F294(O) in cluster B*_Y140 constraint_* was 4.86–6.38 Å (Supplementary Materials, Figure S3D). However, in a few binding solutions, the hydrogen bond interactions between –NH’s of G65 and –COOH of Y140 were sustained.

In the presence of both constraints (hydrophobic region and hydrogen bonding constraint), the tiagabine enantiomers of the respective cluster A*_both constraints_* showed hydrogen bonding of axial –COOH group with –OH of Y140 and –NH’s of G65 (Supplementary Materials, Figure S3E), whereas a few of the tiagabine enantiomers showed a similar hydrogen bonding pattern in cluster B*_both constraints_* to that in cluster A*_both constraints_* (Supplementary Materials, Figure S3F). Moreover, a very marginal interaction between Na1 and axial –COOH enantiomers (cluster A*_both constraints_*) was detected (approximately 3.22–4.20 Å) (Supplementary Materials, Figure S3E). Similarly, the coordination between Na1 and equatorial –COOH enantiomers (cluster B*_both constraints_*) was not plausible as these were at larger distances from each other (3.54–4.81Å) (Supplementary Materials, Figure S3F). Similar to the outputs of the “hydrophobic constraint” (both clusters) and “unconstraint” docking scenarios (cluster A*_unconstraint_*), the carbonyl moiety of F294 in clusters A*_both constraints_* and B*_both constraints_* was observed at a distance of approximately 4.03–5.12Å from the protonated –NH group of tiagabine enantiomers, thereby, representing no interaction (Supplementary Materials, Figure S3E,F). In addition, *S*-configured C–C/S–S clockwise equatorial tiagabine enantiomers (entries 9 and 10) did not endure any of the interactions within the hGAT1 binding pocket (Table 3).

Overall, the selected docked complexes from “unconstraint” docking were further subjected to MD studies on the basis of the following: (1) known hydrogen bond interaction of the protonated –NH group of tiagabine with either F294(O)/S295(O) [25] (Supplementary Materials, Figure S3), which was interrupted in constraint docking scenarios (Table 3), (2) –COOH configuration (i.e., axial or equatorial) of tiagabine enantiomers and respective interactions with Y140, because without implementation of any constraint on the residue Y140, the hydrogen bond interactions with –COOH groups of Tiagabine enantiomers remained preserved. However, the hydrogen bonding constraint on residue Y140 resulted in the loss of coordination with Na1 (Supplementary Materials, Figure S3C,D), which is crucial for antagonists’ binding during the translocation cycle of hGAT1 [26]. Moreover, it has been reported previously that the attachment of bulky groups (i.e., –COOH) in the axial configuration in hGAT1 antagonists is not favorable for interaction [27,28]; therefore, further refinement of tiagabine enantiomers was made on the basis of –COOH configuration (i.e., axial or equatorial) and respective interaction profiles with Y140. According to which, three enantiomers (Table 1, entries 3, 4, and 9) from cluster B*_unconstraint_* possessing equatorial –COOH configurations were selected for further MD simulation study. However, a single enantiomer of tiagabine possessing axial –COOH (Table 1, entry **6**) was also selected from cluster A*_unconstraint_* due to its similar interaction pattern with rest of the three selected enantiomers from cluster B*_unconstraint_*. Moreover, all four selected entries showed already known hydrogen bond interaction between –COOH and –NH of G65. Therefore, a total of four tiagabine enantiomers from the “unconstraint” docking scenario were selected for further MD studies.

### 2.4. Molecular Dynamics of Selected Tiagabine Enantiomers in hGAT1

To explore the stability and conformational flexibility of the selected four tiagabine enantiomers (Table 1: entries 3, 4, 6, and 9) in a complete membrane-aqueous system surrounding, the 100 ns MD simulations using Desmond [29] were performed as described in methods section followed by the determination of C_alpha_ Root Mean Square Deviation (C_α_RMSD) and C_alpha_ Root Mean Square Fluctuation (C_α_RMSF).

**Figure 4:**
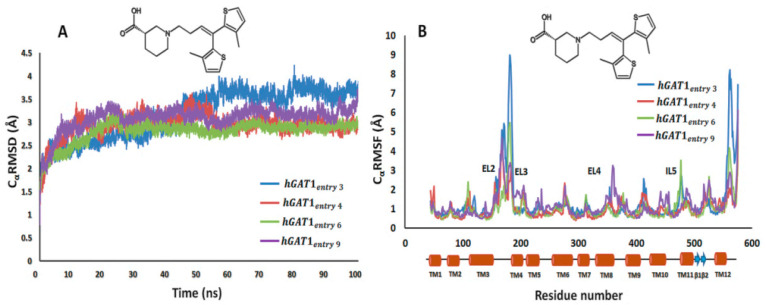
100 ns MD simulations of selected tiagabine enantiomers. (**A**) hGAT1_entry 6_ and hGAT1_entry 4_ enantiomers showed the lowest C_α_RMSD (approx. 2.5 Å) as compared to the entries 3 and 9 of Table 1. (**B**) C_α_RMSF for the fluctuations of individual residues showed significant loss of conformational stability specifically in EL2 region of hGAT1 between TM3 and TM4 (residues number 100 to 200).

The analysis of C_α_RMSD showed that after vigorous fluctuations of 30 ns of MD the simulations remained stabilized for the next 20 ns followed by a fluctuation of 15 ns more (after 50 ns) as shown in Figure 4. However, from ~65 ns of simulation, the C_α_RMSD values of the selected tiagabine enantiomers were maintained in the range of 2.7–4.0 Å. Briefly, the complexes hGAT1_entry 6_ shown in green and hGAT1_entry 4_ shown in brown showed the lowest C_α_RMSD values, i.e., 2.7 Å (Figure 4A); whereas, the complexes hGAT1_entry 9_ shown in purple and hGAT1_entry 3_ shown in blue have displayed a C_α_RMSD of 3.4 and 4.0 Å, respectively (Figure 4A). Moreover, the C_α_RMSD for the fluctuations of individual residues (C_α_RMSF) of all the four complexes generated during the MD simulation was calculated to characterize the mobility of individual residues (shown in Figure 4B) that has depicted the significant loss of conformational stability specifically in extracellular loop 2 (EL2) region of hGAT1 between TM3 and TM4.

**Figure 5:**
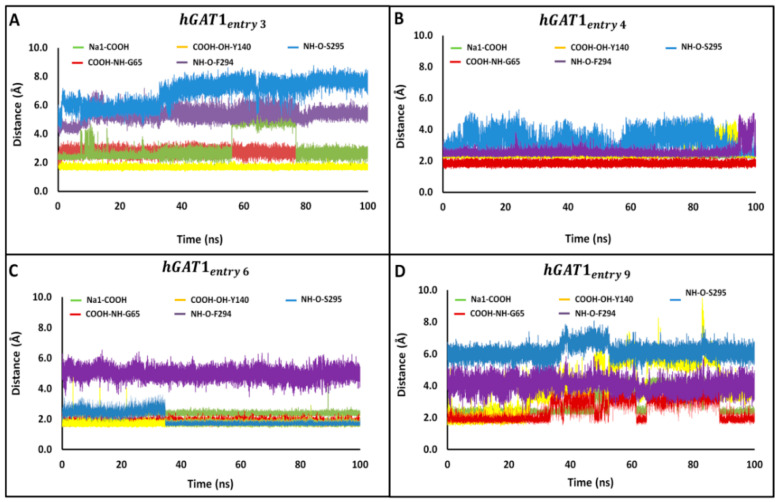
Distances between the tiagabine enantiomers and hGAT1 interaction site residues. Time series are shown for distances between the protonated –NH group of tiagabine and F294(O)/S295(O); and between the average position of –COOH group of tiagabine and G65 (–NH), Y140 (–OH), and Na1 of the entries 3, 4, 6, and 9 representing the complexes (**A**) hGAT1_entry 3_, (**B**) hGAT1_entry 4_, (**C**) hGAT1_entry 6_, and (**D**) hGAT1_entry 9_, respectively, of Table 1.

Time series of distance profiles monitoring tiagabine binding during the MD simulations for the hGAT1–Tiagabine complexes is shown in Figure 5. The pair-wise atomic distances used to monitor tiagabine enantiomers’ binding geometries are between the tertiary –NH group and specific binding site residues F294 (O)/S295(O) and between the –COOH group and binding site residues G65 (–NH), Y140 (–OH), and Na1. The average MD distance between –COOH and Na1 was measured to be 2.4–3.0 Å among all the four complexes (Figure 5). Moreover, the average pair-wise atomic distances between the –COOH group of tiagabine enantiomers and –NH of G65 and –OH of Y140 were 1.8 and 1.7 Å, respectively (Figure 5A–D). The average MD distance between the F294 (O)/S295 (O) and the protonated –NH group of tiagabine enantiomer was 2.6 Å in case of entries 4 and 6 of Table 1 (Figure 5B,C), whereas for entries 3 and 9, an increase in distance, i.e., 5.3 Å with S295 (O) and 4.0 Å with F294 (O) was observed as shown in Figure 5A,D.

**Figure 6:**
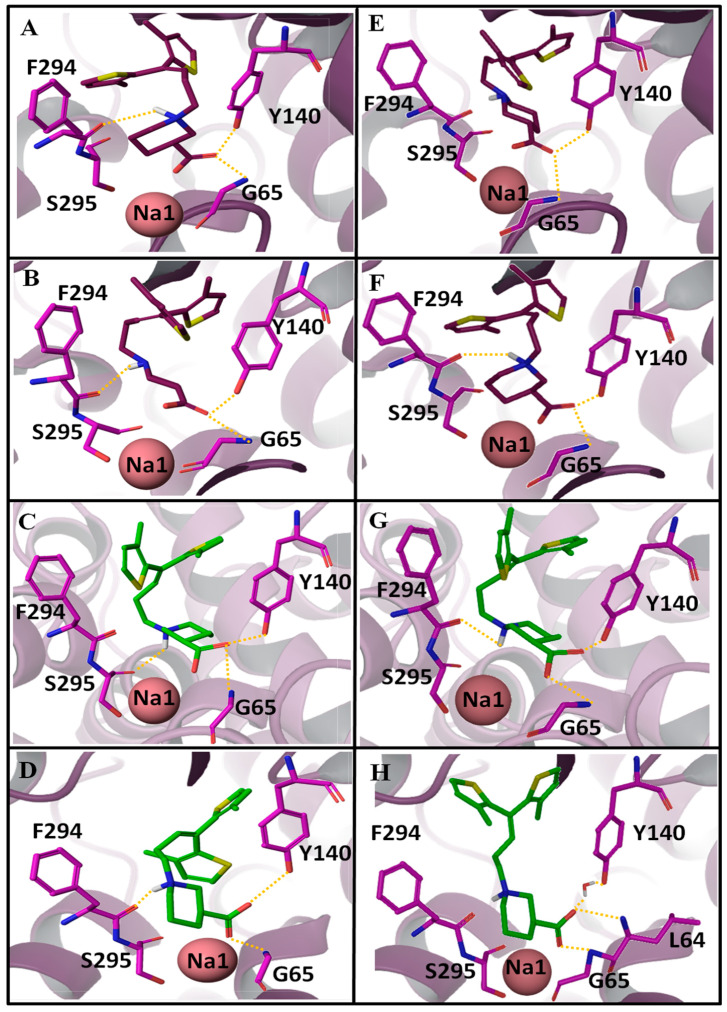
Comparison of ligand-protein interactions in the selected tiagabine enantiomers (Table 1, entries 3, 4, 6, 9). (**A**–**D**) Interaction pattern of entries 3, 4, 6, 9 in the hGAT1 binding pocket embedded in complete membrane-aqueous system at 0 ns and (**E**–**H**) after 100 ns of MD.

To further analyze the ligand–protein interactions, MD simulations of all the four complexes were compared (Table 4). Furthermore, in order to support our results, Figure 6 illustrates before MD (i.e., at 0 ns) and after MD (i.e., at 100 ns) snapshots of all the four complexes. In the case of hGAT1_entry 3_, after 100 ns of MD the interaction of F294 (O) that was observed at 0 ns (Figure 6A) with the *R*-configured protonated –NH group got disrupted (Figure 6E, Table 4). However, in the case of hGAT1_entry 4_, all the interactions remained intact, i.e., involvement of the carbonyl moiety of F294 in hydrogen bond formation with the *R*-configured protonated –NH group of tiagabine and also between –COOH group of tiagabine and –NH of G65, –OH of Y140, and Na1 (Figure 6B,F, Table 4). Interestingly, the interaction pattern of hGAT1_entry 6_ (Figure 6C,G, Table 4) was quite similar to that of hGAT1_entry 4_ (Figure 6F). Skovstrup et al., highlighted the continuous switch of hydrogen bond interaction between the F294 and S295 with the protonated –NH group of tiagabine [25]. Hence, a negligible difference in hydrogen bond interaction of the protonated –NH group was observed in the before (with S295) and after MD (with F294) tiagabine-docked hGAT1 complexes (Figure 6C,G, respectively).

Unlike the other three simulated tiagabine enantiomers (hGAT1_entries 3,4,6_), hGAT1_entry 9_ showed a water-mediated interaction with –OH of Y140. In addition to this, the –COOH group showed hydrogen bonding with the –NH of L64 (unusual) and G65 altogether with its interaction with Na1, however, the interaction between the protonated –NH group and carbonyl moiety of F294 was abolished (Figure 6H). The presence of water molecules in the open-to-out conformation of *Aa*LeuT and dDAT has been previously illustrated in various studies [15,25,30]. For example, in *Aa*LeuT, the bound inhibitor tryptophan has maintained a water-mediated bridge through its –COOH group with the residue tyrosine [30]. In another MD study, the incorporation of a water molecule between –COOH of tiagabine and Y140 resulted in a stable trajectory, which shows that presence of a water molecule does not have any major influence on the ligand protein interaction [25]. However, in dDAT, the direct correlation between the ligand’s –NH group and Na1 was disrupted as both were interconnected through two water molecules, whereas the inhibitor (cocaine)-bound dDAT in its open-to-out conformation lacks the water molecule bridging either with tyrosine or Na1 [15].

Overall, among the four simulated tiagabine enantiomers, hGAT1_entry 4_ and hGAT1_entry 6_ (Table 4) retained the maximum ligand–protein interactions over the course of 100 ns of MD simulation. In addition, the hydrogen bond interactions were observed only between the protonated –NH group of hGAT1_entries 4,6_ of Table 4 with the F294 (O) and S295 (O), respectively. However, the possible reason for the hydrogen bond disruption in the case of hGAT1_entries 3,9_ could be the bending of thiophene rings toward the protonated –NH group as shown in Figure 6E,H thereby hindering the interaction with F294. The MD distances shown in Figure 5 also explain the analysis. Thus, it might be inferred from the MD distances (Figure 5) and ligand–protein interaction profiles (Figure 6) when the sulfur atoms in the thiophene rings are toward the observer’s eye; the tiagabine enantiomers retain the hydrogen bonding with F294 (O)/S295 (O) and adopt the extended conformation in the binding pocket of hGAT1 irrespective of stereoisomerism of the protonated –NH group.

Although –COOH in axial conformation is not auspicious [31], a short MD of 0.012 ns was also run using the respective docked complex as input to determine the reason for the lowest C_α_RMSD of the complex hGAT1_entry 6_ in comparison to hGAT1_entry 4_. It was observed that the axial –COOH group of hGAT1_entry 6_ remained static whereas that of hGAT1_entry 4_ having an equatorial –COOH group was in continuous motion (data not shown). The observed C_α_RMSD for hGAT1_entry 6_ after a short MD of 0.012 ns was about 2.7–3.0 Å approximately, similar to the overall C_α_RMSD maintained throughout 100 ns of MD (Figure 4A). However, in the case of hGAT1_entry 4_, a C_α_RMSD of about 4.0–4.2 Å for the simulation period of 0.012 ns was observed, which gradually decreased to 2.7 Å after 100 ns of MD simulation as shown in Figure 4A. This clearly depicts that the –COOH group attached in axial conformation does not undergo any motion, thus, reflecting a false positive during the 100 ns MD run. Therefore, the hGAT1_entry 4_ was selected as the most probable binding conformation of tiagabine among all the four simulated tiagabine enantiomers.

### 2.5. Cross Validation of hGAT1_entry 4_

**Figure 7:**
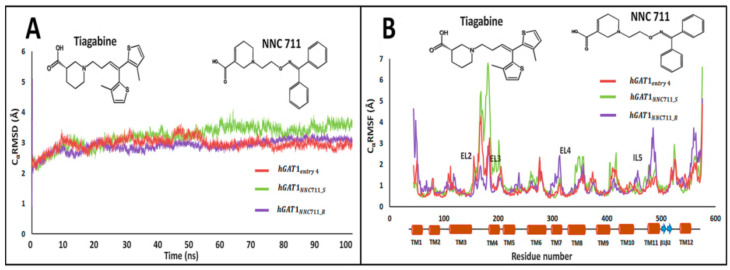
Cross validation of selected tiagabine enantiomer with C_α_RMSD of NNC711 enantiomers through MD simulations. (**A**) hGAT1_NNC711_R_ and hGAT1_entry 4_ complexes showed the lowest C_α_RMSD (approx. 2.5–2.7 Å) (**B**) C_α_RMSF for the fluctuations of individual residues showed the significant loss of conformational stability specifically in EL2 region of hGAT1 (between TM3 and TM4, residue number 100 to 200).

The final tiagabine conformation, i.e., hGAT1_entry 4_, was cross validated by binding conformation of two enantiomeric complexes including hGAT1_NNC711_R_ and hGAT1_NNC711_S_ of another already known inhibitor of GAT1, i.e., NNC711 (0.040 µM) [32,33]. The C_α_RMSD of hGAT1_NNC711_R_ was observed to be similar to that of the hGAT1_entry 4_, i.e., approximately 2.7 Å (Figure 7A). Similarly, the C_α_RMSD of the individual residues (RMSF) for docked NNC711 was in agreement with that of the hGAT1_entry 4_ (Figure 7B). Hence, this validates that tiagabine with the protonated –NH group in its *R*-configuration along with the –COOH group in equatorial configuration is more promising in terms of interaction within the hGAT1 binding pocket.

Moreover, our results could be further strengthened by comparing the interaction pattern of the hGAT1_entry 4_ with the Skovstrup’s interactions analysis of constraint *cis* (*R*) enantiomer of tiagabine that showed hydrogen bond interaction between the protonated –NH group of tiagabine and carbonyl oxygen moiety of F294, however, the constraint *trans* (*S*) enantiomer was unable to sustain such interaction [25].

Furthermore, during the course of 100 ns MD simulation of the hGAT1_entry 4_ complex, the water-mediated interaction between the protonated –NH group of tiagabine and F294 was observed during 5–35 ns. Afterward, the protonated –NH group and F294 remained in direct contact with each other. However, the binding pocket remained occluded with four water molecules throughout the simulation. Hitherto, Skovstrup et al. have conducted an MD study of hGAT1 for about 24 ns and observed a water-mediated interaction between protonated the –NH group of tiagabine and carbonyl oxygen of F294 [25]. However, no such interaction has been observed in the crystal structure of the cocaine-bound transporter, dDAT [15]. Until 35 ns of simulation, our result for the hGAT1_entry 4_ is in accordance with the Skovstrup et al. analysis, however, tiagabine mediated the direct hydrogen bond interaction between the protonated –NH group and F294 for the rest of the 65 ns. Thus, it might be factual to state that a long simulation run could provide more promising results in terms of stability and flexibility of the protein–ligand complexes.

## 3. Materials and Methods

### 3.1. Ligand Preparation

Stereoisomers of tiagabine constituting (i) *R*-configured –NH group and (ii) *S*-configured –NH group were generated followed by protonation, desalination, and tautomerization at pH 7.0 ± 2.0 using LigPrep in Schrodinger modeling package [34]. Later, energy minimization of the stereoisomers was carried out using the OPLS 2005 force field while retaining the specified chiralities of the input maestro file.

### 3.2. Protein Preparation and Molecular Docking Studies

#### 3.2.1. Docking Protocol I (Unconstraint Docking Protocol)

An in-house preprocessed hGAT1 model built in open-to-out conformation [35] was used to dock both *R*- and *S*-configured tiagabine. Briefly, homology modeling of hGAT1 (UniProt: P30531) in open-to-out conformation was based on the X-ray crystallographic structure of the *Drosophila melanogaster* dopamine transporter (dDAT) (Protein Data Bank ID: 4XP4) with the bound cocaine substrate and the co-transport di-sodium/chloride (2Na^+^/1Cl^−^) ions within its structurally conserved ion/substrate binding sites. The binding pocket was identified by generating the receptor (hGAT1) grid using the default parameters, i.e., charge cutoff 0.25, van der Waals scaling factor 1.0, and OPLS 2005 force field followed by generation of a cubic box (17 × 17 × 17 Å) around the centroid (X: −0.86, Y: 3.96, Z: −0.24) of already known active site residues (G59-G63, G65-W68, L136, Y139, Y140, I143, Q291-G297, L300, N327, D395, S396, T400 [14,36,37]) using Glide [38,39,40]. Further, the standard precision (SP) method with a partial charge cutoff 0.15 and van der Waals scaling factor of 0.80 was applied to generate 20 poses per *R*- and *S*-enantiomer of tiagabine in Schrodinger-Maestro [38,39] using the Glide score (G_score_) [41] as a fitness function, as can be seen in Equation (1):

(1) Gscore=0.065*vdW+0.130*Coul+Lipo+HB+Metal+BuryP+RotB+Site

**Figure 8:**
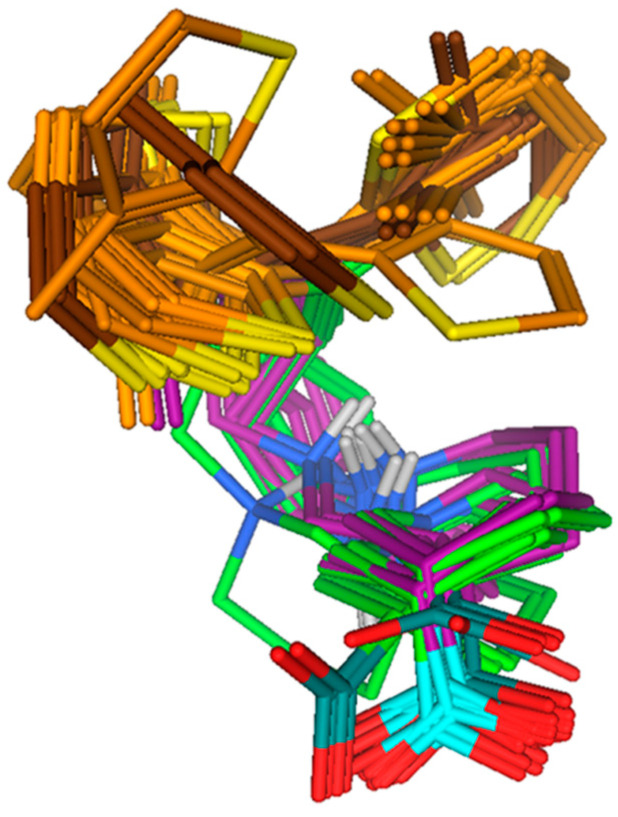
The output of docking protocol I representing conformational overlap of *R*- and *S*-enantiomers of tiagabine. Axial and equatorial –COOH groups are highlighted with light and dark cyan, whereas clockwise and anticlockwise orientations of thiophene rings are displayed in light and dark brown. Tiagabine enantiomers with *S*-configured –NH groups are represented in green, while *R*-configured groups are in maroon.

The output of docking protocol I representing the binding poses of tiagabine in different configuration states at (iii) axial –COOH (iv) equatorial –COOH (v) clockwise thiophene rings, and (vi) counterclockwise thiophene rings were energy minimized using OPLS 2005 force field followed by ligand–protein interaction analysis of respective complexes. The overall binding position of 3/4 enantiomers was similar in the hGAT1 binding pocket with a negligible difference in the binding interaction as shown in Figure 8. Therefore, a single representative of each configurational state out of cluster of poses exhibiting similar binding position, interaction, and having the lowest G_score_ value was selected for docking protocol II as well. Additionally, some of the binding poses having different binding positions were also selected for docking protocol II and for further ligand–protein interaction analysis. Docking protocol II, utilizing the screened tiagabine enantiomers from docking protocol I, was performed to nullify the previous hypothesis that molecular docking of ligands inside hGAT1 is only possible when constraint is applied on the binding site residues [27].

#### 3.2.2. Docking Protocol II (Constraint Docking Protocol)

Various reports highlighted the crucial role of Y140 in the GABA-induced translocation through hGAT1 by mediating hydrogen bond interaction with the –COOH group of GABA [35,42]. Therefore, constraint docking on Y140 residue has been performed previously to delineate the molecular basis of ligand–transporter interactions of hGAT1 antagonists [25,27]. Hence, in order to remove any bias in docking protocol I, we performed constraint docking on the finally selected binding poses of docking protocol I. Our applied constraints include the following: (i) hydrogen bonding constraint on Y140 to form hydrogen bond interaction with –COOH of tiagabine, (ii) hydrophobic region constraint on the thiophene rings, and (iii) application of both hydrogen bonding and hydrophobic region constraints on the tiagabine enantiomers. Briefly, 20 poses per *R*- and *S*-conformation of tiagabine in each constraint situation were generated. The constraint docking poses showed similar binding position as that of the selected 10 unconstraint docking poses from protocol I. Out of the constraint and unconstraint binding solutions of *R*- and *S*-tiagabine, only those binding conformations that sustained already reported interaction within the binding pocket of hGAT1 were selected for further molecular dynamics (MD) simulation. The major goal of MD simulations was to assess the stability of the respective ligand–transporter complexes. Overall, the proposed methodology for the identification of the most stable tiagabine conformation for hGAT1 transport reuptake inhibition is shown in Supplementary Materials, Figure S1.

### 3.3. Molecular Dynamics Studies

The final selected hGAT1–tiagabine complexes on the basis of ligand–protein interactions profile in hGAT1 from different conformational tiagabine enantiomers were further refined with a restraint energy minimization OPLS 2005 force field under an implicit generalized Born solvent model to remove all steric clashes. Each of the selected complexes was embedded with a 15 Å buffer region from the edge of the complex within a phosphatidylcholine (POPC) lipid membrane. In addition, the complexes were also embedded with an explicit transferable intermolecular potential with 3 points (TIP3P) water layer at 0.15 M NaCl salt concentration as counterions inside an orthorhombic box. MD simulation for each of the selected tiagabine conformers was carried out using Desmond with a default initialization protocol, followed by 100 ns unrestraint production simulation run under constant-area isothermal isobaric (NPAT) conditions at 300 K and 1 atm. The stability of each of the hGAT1–tiagabine complex was assessed by evaluating the C_α_RMSD and C_α_RMSF with respect to the minimized starting structure (Supplementary Materials, Figure S1). The most stable tiagabine conformations were further compared with the most stable (100 ns MD run) *R*- and *S*-configurations of another known inhibitor of GAT1; NNC711 (0.040 µM) [33] to select the optimal binding solution from the tiagabine conformations.

## 4. Conclusions

The tiagabine stereoisomer having a protonated –NH group in the *R*-conformation and an equatorial –COOH group has been identified as most probable binding conformation within hGAT1 on the basis of the ligand–protein interaction profile and hydrogen bond stability analysis, i.e., hGAT1_entry 4_. In order to further strengthen our result in terms of conformational stability in the hGAT1 binding pocket, cross validation with another known inhibitor NNC711 was performed. The finally selected tiagabine enantiomer (hGAT1_entry 4_) may serve as a reference binding conformation in virtual screening followed by experimental validation for the quest of new hGAT1-selective antiepileptic agents.

## Figures and Tables

**Table 1:** *R*- and *S*-configured tiagabine enantiomers and the associated terminology for thiophene rings and –COOH group of the piperidine ring. The highlighted entries were selected for molecular dynamics (MD) simulations on the basis of ligand–protein interactions in hGAT1 binding pocket.

Configuration of Protonated N Atom	Direction of Thiophene Rings with Respect to the Observer’s Eye	Orientation of Thiophene Rings	–COOH Configuration	Terminology	Entry Code
*R* (maroon purple)	C–C	Clockwise	Axial	*R*-configured C–C clockwise axial	1
	S–S			*R*-configured S–S clockwise axial	2
	C–C	Counterclockwise	Equatorial	*R*-configured C–C counterclockwise equatorial	3
	S–S			*R*-configured S–S counterclockwise equatorial	4
*S* (green)	C–C	Clockwise	Axial	*S*-configured C–C clockwise axial	5
	S–S			*S*-configured S–S clockwise axial	6
	C–C	Counterclockwise	Equatorial	*S*-configured C–C counterclockwise equatorial	7
	S–S			*S*-configured S–S counterclockwise equatorial	8
*S* (green)	C–C	Clockwise	Equatorial	*S*-configured C–C clockwise equatorial	9
	S–S			*S*-configured S–S clockwise equatorial	10

**Table 2:** Top G_score_ values of selected tiagabine enantiomers in hGAT1 from unconstraint and constraint docking.

Code	Entries Taken from Table 1	G_score_ (Kcal/mol)			
		Unconstraint	Hydrophobic Region Constraint	Hydrogen Bonding Constraint	Hydrophobic and Hydrogen Bonding Constraints
1	*R*-configured C–C clockwise axial	−7.13	−7.00	−7.37	−7.37
2	*R*-configured S–S clockwise axial	−7.09	−5.25	−7.38	−5.25
3	*R*-configured C–C counterclockwise equatorial	−7.73	−9.36	−6.86	−6.46
4	*R*-configured S–S counterclockwise equatorial	−7.98	−6.21	−6.43	−6.83
5	*S*-configured C–C clockwise axial	−6.97	−7.7	−7.07	−6.35
6	*S*-configured S–S clockwise axial	−8.08	−8.14	−5.53	−6.39
7	*S*-configured C–C counterclockwise equatorial	−7.00	−7.16	−6.86	−6.20
8	*S*-configured S–S counterclockwise equatorial	−6.15	−5.83	−6.22	−5.37
9	*S*-configured C–C clockwise equatorial	−7.70	−6.58	−5.91	−5.91
10	*S*-configured S–S clockwise equatorial	−6.91	−7.00	−5.53	−5.68

**Table 3:** Distances range of –COOH and –NH groups in binding solutions of *R*- and *S*-enantiomers of tiagabine in cluster A, B, and additional two entries of *S*-configured C–C/S–S clockwise equatorial tiagabine enantiomers from amino acid residues of the hGAT1 binding pocket under different docking scenarios.

			Cluster A		Cluster B		*S*-Configured C–C/S–S Clockwise Equatorial Tiagabine Enantiomers	
		GAT1 Residues	Tiagabine’s –COOH Group (Å)	Tiagabine’s –NH Group (Å)	Tiagabine’s –COOH Group (Å)	Tiagabine’s –NH Group (Å)	Tiagabine’s –COOH Group (Å)	Tiagabine’s –NH Group (Å)
**Unconstraint**		G65 (–NH)	1.8	-	1.8	-	1.8–3.9	-
		Y140 (–OH)	3.7–4.0	-	1.8–3.5	-	1.8–5.7	-
		F294 (O)	-	-	-	1.8–4.0	-	1.8–4.2
		S295 (O)	-	1.8–3.6	-	-	-	-
		Na1	2.3–2.4	-	2.5–2.6	-	2.6–4.05	-
**Constraint**	Hydrophobic region constraint	G65 (–NH)	1.8	-	1.8	-	1.8	-
		Y140 (–OH)	4.0–4.4	-	2.4–3.0	-	4.2–4.5	-
		F294 (O)	-	-	-	3.8–3.9	-	3.6–3.8
		S295 (O)	-	3.2–3.5	-	-	-	-
		Na1	2.3–2.6	-	2.3–2.6	-	2.5–3.2	-
						
	Hydrogen bonding constraint	G65 (–NH)	1.8	-	1.8	-	2.2–2.4	-
		Y140 (–OH)	1.8	-	1.8	-	1.8	-
		F294 (O)	-	-	-	4.8–6.3	-	4.6–5.2
		S295 (O)	-	4.8–5.1	-	-	-	-
		Na1	3.2–6.0	-	3.2–5.9	-	3.2–4.8	-
						
	Hydrophobic region and hydrogen bonding constraints	G65 (–NH)	1.8	-	1.8	-	3.8–4.2	-
		Y140 (–OH)	1.8	-	1.8	-	3.2	-
		F294 (O)	-	4.0–5.1	-	4.0–5.0	4.2–4.6	-
		S295 (O)	-	-	-	-	-	-
		Na1	3.2–4.2	-	3.5–4.8	-	3.2–4.6	-

**Table 4:** Ligand–protein interaction profiles of the selected hGAT1–tiagabine complexes before and after 100 ns of MD simulations.

		Before MD		After MD	
	hGAT1 Residues	Tiagabine’s –COOH Group	Tiagabine’s –NH Group	Tiagabine’s –COOH Group	Tiagabine’s –NH Group
hGAT1_entry 3_	G65(–NH)	1.8 Å	-	1.8 Å	-
	Y140 (–OH)	1.8 Å	-	1.8 Å	-
	F294 (O)	-	1.8 Å	-	3.2 Å
	S295 (O)	-	-	-	-
	Na1	2.4 Å	-	2.3 Å	-
hGAT1_entry 4_	G65(–NH)	1.8 Å	-	1.8 Å	-
	Y140 (–OH)	1.8 Å	-	1.8 Å	-
	F294 (O)	-	1.8 Å	-	1.8 Å
	S295 (O)	-	-	-	-
	Na1	2.3 Å	-	2.4 Å	-
hGAT1_entry 6_	G65(–NH)	1.8 Å	-	1.8 Å	-
	Y140 (–OH)	1.8 Å	-	1.8 Å	-
	F294 (O)	-	-	-	1.8 Å
	S295 (O)	-	1.8 Å	-	-
	Na1	2.3 Å	-	2.2 Å	-
hGAT1_entry 9_	G65(–NH)	1.8 Å	-	1.8 Å	-
	Y140 (–OH)	1.8 Å	-	5.2 Å	-
	F294 (O)	-	1.8 Å	-	4.6 Å
	S295 (O)	-	-	-	-
	Na1	2.4 Å	-	2.3 Å	-

## Supplementary Materials

Supplementary materials are available online. Figure S1. Workflow of selection of stable docking pose of hGAT1-Tiagabine complex followed by MD studies; Figure S2. Unconstraint docking of *R*- and *S*-enantiomers of tiagabine in hGAT1. Ligand protein interactions of tiagabine enantiomers of (**A**) cluster A_unconstraint_ and (**B**) cluster B_unconstraint_ in hGAT1. (**C**) The more pronounce deviation of equatorial –COOH from Y140 (5.7Å) was observed in the *S*-configured S-S clockwise equatorial unconstraint enantiomer of tiagabine (Table 1, entry 10); Figure S3. Application of hydrophobic constraint, Y140 constraint and both hydrophobic and hydrogen bond with Y140 constraint in docking of tiagabine enantiomers within hGAT1 binding pocket. (**A**) Distances of protonated –NH group and axial –COOH from S295 (3.21–3.50Å) and –OH of Y140 (4.43Å), respectively in enantiomers of cluster A_hydrophobic constraint_, (**B**) Distances of protonated –NH group and equatorial –COOH of tiagabine enantiomers from F294 (3.84–3.99Å) and –OH of Y140 (2.46–3.09Å), respectively in cluster B_hydrophobic constraint_. (**C**, **D**) Hydrogen bonding between OH of Y140 and few of the –NHs of G65 with –COOH groups of tiagabine enantiomers in both clusters (1 and 2 of Y140 constraint) was observed. (**E**, **F**) Lack of coordination between Na1 and –COOH groups in clusters A_both constraints_ and B_both constraints_ was observed due to increased distance (3.22–4.8Å). Interaction between F294 and protonated –NH group was also disrupted.
