# Supplementary material for: Molecular Dynamic Simulations to Probe Stereoselectivity of Tiagabine Binding with Human GAT1
Source: Molecules. 2020 Oct 16;25(20):4745. doi: 10.3390/molecules25204745 (PMC7587590; doi:10.3390/molecules25204745)
Supplement: Supplementary file 1 [file molecules-25-04745-s001.pdf]

# Molecular Dynamic Simulations to Probe Stereoselectivity of Tiagabine Binding with Human GAT1

**Sadia Zafar and Ishrat Jabeen ***

Research Center for Modeling and Simulation (RCMS), National University of Sciences and Technology (NUST), Islamabad 44000, Pakistan; sadia.zafar@rcms.nust.edu.pk
* Correspondence: ishrat.jabeen@rcms.nust.edu.pk

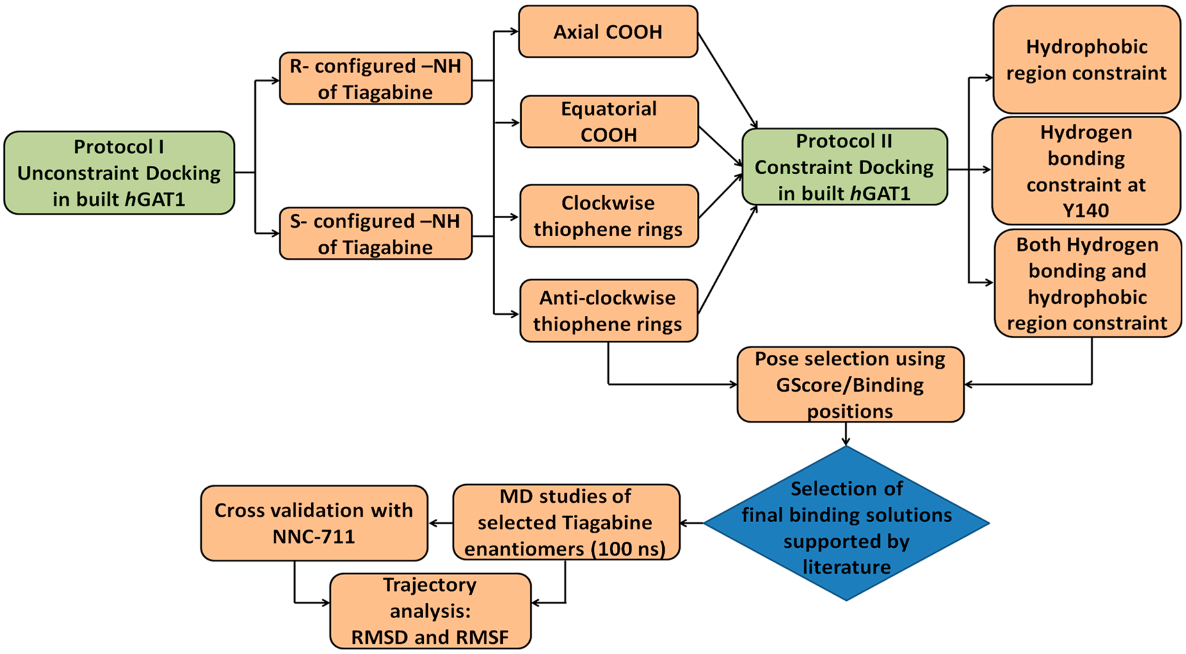


**Figure 1.** Workflow of selection of stable docking pose of hGAT1—Tiagabine complex followed by MD studies.

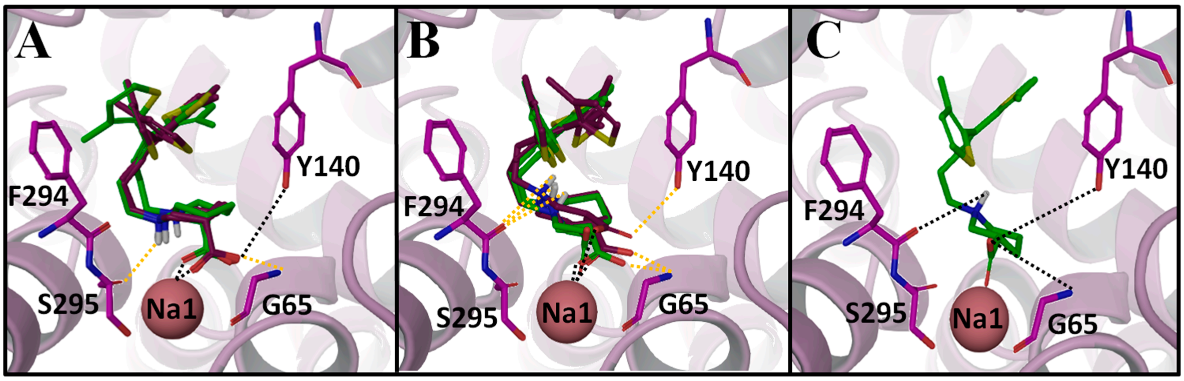


**Figure 2.** Unconstraint docking of *R*- and *S*-enantiomers of tiagabine in hGAT1. Ligand protein interactions of tiagabine enantiomers of (**A**) cluster $A_{unconstraint}$ and (**B**) cluster $B_{unconstraint}$ in hGAT1. (**C**) The more pronounce deviation of equatorial –COOH from Y140 (5.7Å) was observed in the *S*-configured S-S clockwise equatorial unconstraint enantiomer of tiagabine (Table 1, entry 10).

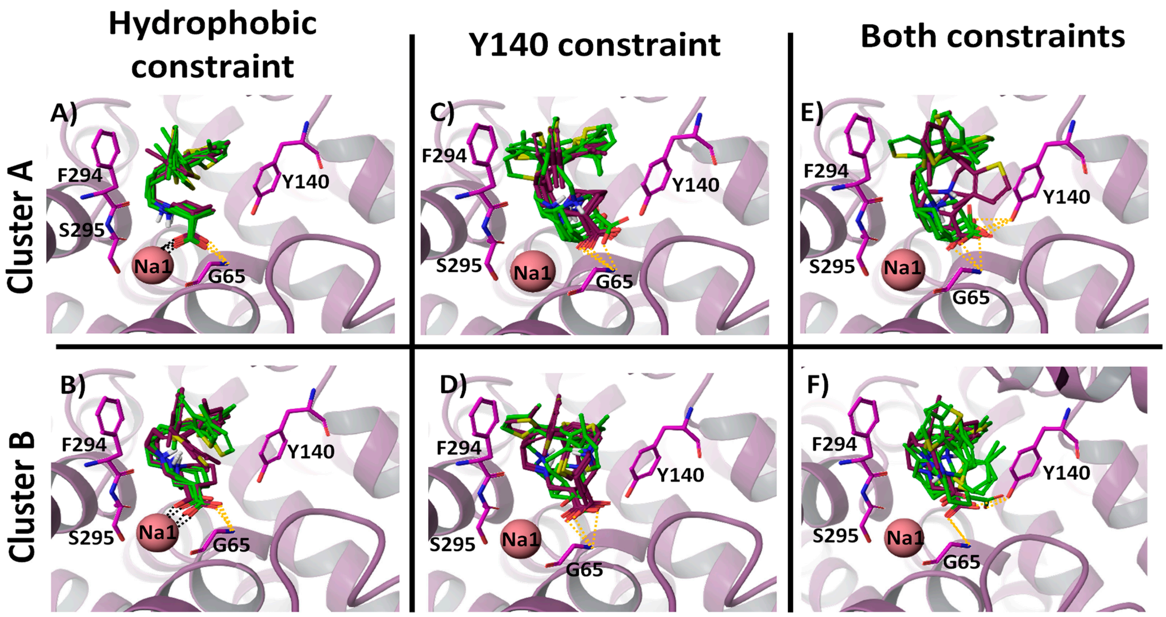


**Figure 3.** Application of hydrophobic constraint, Y140 constraint and both hydrophobic and hydrogen bond with Y140 constraint in docking of tiagabine enantiomers within hGAT1 binding pocket. (**A**) Distances of protonated –NH group and axial –COOH from S295 (3.21–3.50Å) and –OH of Y140 (4.43Å), respectively in enantiomers of cluster $A_{hydrophobic\ constraint}$, (**B**) Distances of protonated –NH group and equatorial –COOH of tiagabine enantiomers from F294 (3.84–3.99Å) and –OH of Y140 (2.46–3.09Å), respectively in cluster $B_{hydrophobic\ constraint}$. (**C,D**) Hydrogen bonding between OH of Y140 and few of the –NHs of G65 with –COOH groups of tiagabine enantiomers in both clusters (1 and 2 of Y140 constraint) was observed. (**E,F**) Lack of coordination between Na1 and –COOH groups in clusters $A_{both\ constraints}$ and $B_{both\ constraints}$ was observed due to increased distance (3.22–4.8Å). Interaction between F294 and protonated –NH group was also disrupted.
